# Transcriptomic analysis of *Crassostrea sikamea* × *Crassostrea angulata* hybrids in response to low salinity stress

**DOI:** 10.1371/journal.pone.0171483

**Published:** 2017-02-09

**Authors:** Lulu Yan, Jiaqi Su, Zhaoping Wang, Xiwu Yan, Ruihai Yu, Peizhen Ma, Yangchun Li, Junpeng Du

**Affiliations:** 1 Fisheries College, Ocean University of China, Qingdao, Shandong, China; 2 The Key Lab of South China Sea Fishery Resources Exploitation & Utilization, Ministry of Agriculture, South China Sea Fisheries Research Institute, Chinese Academy of Fishery Sciences, Guangzhou, Guangdong, China; 3 Engineering Research Center of Shellfish Culture and Breeding of Liaoning Province, College of Fisheries and Life Science, Dalian Ocean University, Dalian, Liaoning, China; Xiamen University, CHINA

## Abstract

Hybrid oysters often show heterosis in growth rate, weight, survival and adaptability to extremes of salinity. Oysters have also been used as model organisms to study the evolution of host-defense system. To gain comprehensive knowledge about various physiological processes in hybrid oysters under low salinity stress, we performed transcriptomic analysis of gill tissue of *Crassostrea sikamea* ♀ × *Crassostrea angulata*♂ hybrid using the deep-sequencing platform Illumina HiSeq. We exploited the high-throughput technique to delineate differentially expressed genes (DEGs) in oysters maintained in hypotonic conditions. A total of 199,391 high quality unigenes, with average length of 644 bp, were generated. Of these 35 and 31 genes showed up- and down-regulation, respectively. Functional categorization and pathway analysis of these DEGs revealed enrichment for immune mechanism, apoptosis, energy metabolism and osmoregulation under low salinity stress. The expression patterns of 41 DEGs in hybrids and their parental species were further analyzed by quantitative real-time PCR (qRT-PCR). This study will serve as a platform for subsequent gene expression analysis regarding environmental stress. Our findings will also provide valuable information about gene expression to better understand the immune mechanism, apoptosis, energy metabolism and osmoregulation in hybrid oysters under low salinity stress.

## Introduction

Hybridization enhances genetic variance, allowing ecesis of unexploited niches. Following recombination, ‘transgressive’ quantitative variation is effected resulting in more extreme traits than either of the parents. Therefore, hybridization not only plays an important role in speciation but also forms the backdrop for evolutionary innovations [1]. Hybridization breeding, which is defined as the mating of animals from different species, strains or inbred lines, facilitates genetic rearrangements, which may have strong selective value in aquaculture [2, 3]. There are several instances where the resulting hybrid offsprings show hybrid vigor or heterosis in growth rate, weight, survival and adaptability to extremes of temperature, salinity, etc. [4–8]. Recently, many attempts have been made at hybridization between various *Crassostrea* species. For instance, successful fertilization has been reported for the following crosses amongst others- *C*. *hongkongensis × C*. *ariakensis* [9], *C*. *ariakensis ×C*. *angulata* [10], *C*. *ariakensis × C*. *sikamea* [11], and *C*. *angulata × C*. *sikamea* [12]. Previous studies have mostly focused on the survival and growth rate of these hybrids under various environmental conditions [9–12].

Salinity is one of the environmental factors which directly affect the survival, growth, and physiological function of oysters [13–16]. Oysters are generally euryhaline mollusks, although the preferred salinity range varies from species to species [11]. The Kumamoto oyster (*C*. *sikamea*) and the Portuguese oyster (*C*. *angulata*) have different preferences of optimum salinities, although they naturally coexist along the southern coast of China. Adult *C*. *angulata* oyster (AA) can survive under salinities ranging from 10 ppt to 45 ppt, however the optimum salinity for this species varies from 26 ppt to 35 ppt in adult stage [17]. *C*. *sikamea* (SS) can survive under salinities ranging from 8 ppt to 35 ppt in the larval stage [18]. However, the salinity preference of SS adults is unknown. The salinity range of the natural habitat where SS oysters are usually found ranges from 7 to 34.2 ppt [18, 19]. The survival and growth of hybrid oysters are affected by different salinities [9, 11, 20]. The optimal salinity of hybrid oyster depends on the parental species, but it is usually different from the optimal salinities of the parents. Studies from our lab has shown that AA larvae could survive in salinity as low as 20 ppt, while SS and SA larvae could survive in 15 ppt [20]. On the other hand, the survival and metamorphosis of the hybrid oysters (SA) were higher than those of two inbred parental groups (SS and AA) under low salinity stress, with obvious hybrid vigor during the larvae stage [20]. However, the molecular mechanism of heterosis in hybrid oysters under low salinity stress remains largely unknown.

There is periodic fluctuation of salinity in the natural habitat of oysters due to tidal cycle, rainfall and drainage from adjacent territorial sites [14]. Such fluctuations can further affect the physiological function of oysters. Low/high salinity stress can decrease/increase the pallial cavity salinity and haemolymph potassium and sodium concentration of *C*. *gigas* [21]. An in-vitro decrease of salinity was associated with haemocyte mortality in *C*. *gigas* [22]. Previous reports demonstrate the combined effects of temperature and salinity on oxygen consumption in mollusks. At a temperature of 25°C, low salinity could enhance oxygen consumption in *C*. *virginica* and *Ostrea edutis* [23, 24]. Oysters exhibit a number of biochemical mechanisms that control cellular osmolality following salinity stress, such as an increase or decrease of certain free amino acids or that of the glycine betaine [25, 26]. Salinity may also affect the immune system of oysters as part of its general effects on physiology [27]. Low salinity can decrease the activity of phenol oxidase which is a key component of the immune system [28]. Previous studies indicate that low salinity also alters the expression levels of proteins and genes involved in immune responses in *C*. *gigas* [25, 29]. Furthermore, both laboratory and field studies have demonstrated a correlation between changes in salinity level and infection in bivalves [30]. Even though oysters lack an adaptive immune system, they can flourish in microbe-rich estuaries as filter-feeders. This unique feature makes oysters interesting models to study the evolution of host-defense system [27].

Heterosis, also known as hybrid vigour, is widespread in plants and animals. In spite of its ubiquitous presence in nature, the molecular basis of this phenomenon remains elusive [31]. Here we present a transcriptomic study of *C*. *sikamea* (SS) ♀ × *C*. *angulata* (AA) ♂ hybrids (SA) under low salinity stress. High throughput Illumina RNAseq technology was employed to compare the relative gene expression levels between hybrid oysters maintained in low salinity (treatment) and natural seawater (control). Sample libraries were prepared from RNA extracted from the gills of the organism. This study provides an insight into the genes underlying tolerance of the hybrid oyster to a hypo-osmotic environment. Furthermore, it also facilitates further analysis of the immune mechanisms in shellfish hybrids, under low salinity stress.

## Materials and methods

### Ethics statement

Oysters handling was conducted in accordance with the guidelines and regulations established by the Ocean University of China and the local government.

### Experimental study design

One year old *C*. *sikamea* (SS), *C*. *angulata* (AA) and hybrids (SA) oysters were procured from the oyster farm located in Beihai, Guangxi, China. The oysters were acclimated in tanks (40 cm × 50 cm × 60 cm) containing aerated filtered seawater (salinity 33.5 ± 0.3 ppt, pH 8.03 ± 0.03, 25 ± 0.5°C) for two weeks prior to experiments. Twenty oysters were individually tagged as SS, SA and AA, and were divided into two groups in separate tanks (control and treatment). For the treatment group, a low salinity level of 10 ppt was achieved by reducing the salinity by 2 ppt per hour by continuous addition of freshwater into the seawater. The setup was maintained for 16 h to induce low salinity stress. The control group was kept under optimal salinity condition by using filtered natural seawater (33.5 ppt). The temperature and PH (25 ± 0.5°C; pH 8.03 ± 0.03) were kept constant to facilitate the process of acclimation. Gills from three SA oysters from both treatment and control groups were collected for further analysis. For qRT-PCR, RNA was extracted from the gills of SS, SA and AA oysters. All sample collections were performed in RNA-Locker (Sangon Biotech, Shanghai, China) and the RNA samples were stored at -80°C for future use.

### RNA extraction and library preparation for transcriptomic analysis

Total RNA was extracted using Trizol reagent and treated with RNase-free DNase I. RNA degradation and contamination was monitored on 1% agarose gel. Purity of the RNA was checked using NanoPhotometer^®^ spectrophotometer (IMPLEN, California, USA). RNA concentration was measured using Qubit^®^ RNA Assay Kit in Qubit^®^ 2.0 Flurometer (LifeTechnologies, California, USA). RNA integrity was assessed using the RNA Nano 6000 Assay Kit and the Agilent Bioanalyzer 2100 system (Agilent Technologies, California, USA).

A total of 1.5 μg RNA per sample was used for the library preparations. Sequencing libraries were generated using NEBNext^®^ Ultra^™^ RNA Library Prep Kit from Illumina^®^ (NEB, New England, USA). Briefly, mRNA was isolated from the total RNA using poly-d(T) oligo-attached magnetic beads. Fragmentation was carried out using divalent cations under elevated temperature followed by synthesis of first and second strand of cDNA. In order to select cDNA fragments (preferentially 150~200 bp in length), the library fragments were purified with AMPure XP system (Beckman Coulter, Beverly, USA) followed by 3 μl USER Enzyme (NEB, New England, USA) treatment. Thereafter, PCR was performed with Phusion High-Fidelity DNA polymerase, Universal PCR primers and Index (X) Primer. The PCR products were purified using AMPure XP system and library quality was assessed by Agilent Bioanalyzer 2100 system. The paired-end RNA-Seq library was sequenced with Illumina HiSeq (2 × 150 bp read length) platform.

### Sequencing data analysis and functional annotation

Using custom perl scripts, reads containing adapter, ploy-N and low quality reads were removed from the raw reads. These reads were then used to perform a *de-novo* transcriptome assembly with Trinity [32]. All the assembled transcripts were matched against the NCBI protein non redundant (nr), Swiss-Prot and Pfam (Protein family) databases to identify the proteins. The E-value cut-off set for the three databases were 1.0 × 10^−5^, 1.0 × 10^−5^ and 0.01 respectively. BLAST2GO [33] was used to identify the GO annotations of uniquely assembled transcripts involved in biological processes, molecular functions and cellular components. A metabolic pathway analysis was performed using the Kyoto encyclopedia of genes and genomes (KEGG).

### Differential expression analysis and functional enrichment

RPKM (Reads Per Kilo bases per Million reads) was used to identify differentially expressed genes between the treatment and the control samples [34]. DEGs were identified by the DESeq2 R package (version V2). Corrected *p*-value of <0.05 and the absolute value of log_2_ fold change ≥ 1 were set as the criteria for significant differential expression.

For pathway enrichment analysis, all DEGs were mapped with the terms of the KEGG database. The statistical enrichment of differentially expressed genes in KEGG pathway was tested by KOBAS software [35]. Gene Ontology (GO) enrichment analysis of the DEGs was carried out using the GOseq R package. This package uses Wallenius non-central hyper-geometric distribution [36] and can adjust for gene length bias in DEGs.

### Expression analysis by qRT-PCR

52 annotated genes were further analyzed by qRT-PCR. Gill samples maintained under low salinity stress from SS, SA and AA were used. Total RNA was extracted using TRIzol^®^ Reagent (Thermo Scientific^™^). First-strand cDNA was synthesized from 900 ng of total RNA using PrimeScript RT Reagent Kit (TaKaRa, Dalian, China) according to the manufacturer's instruction. The cDNA specific primers were designed using AlleleID 6.0 software (Table 1). 28S ribosomal protein S5 (RPS5), Elongation factor 1 alpha (EF1α) and Elongation factor 1 beta (EF1β) were chosen as the housekeeping genes for SS, SA and AA respectively. This is based on our investigation on the use of proper reference genes for SS, SA and AA under the low salinity condition (unpublished work). Each pair of primers was tested to ensure their compatibility with qRT-PCR for the three experimental groups. The qRT-PCR was carried out in the LightCycler^®^ 480 using SYBR^®^ Premix Ex Taq^™^ II (TliRNaseH Plus) (TaKaRa, Dalian, China). The amplifications were performed in a 96-well microtiter plate in a final reaction volume of 20 μl containing 10 μl of SYBR Premix Ex Taq II (TliRNaseH Plus)(2×), 0.8 μl (each) gene-specific forward and reverse primers (10 μM), 6.4 μl RNase-free water and 2.0 μl cDNA (< 100 ng). PCR reaction conditions were as follows: 95°C for 30 s followed by 40 cycles of 95°C for 5 s, 55°C for 30 s and 72°C for 30 s. Additionally, formation of a single product was verified for all the tested genes using melting curve analysis.

**Table 1:** Primer sequences for amplification of target and reference genes in three different species.

gene_id	species	Sense Primer (5'-3')	Anti-sense Primer (5'-3')
**c140212_g1**	SS/SA/AA	AAGGAAGTGGCAGCAATCTC	GTTCTACACATCTGTCTACATAAGG
**c148773_g1**	SS/SA/AA	TCTTTCTTCTACATCTGCGTTTG	GTTCCTCATCCTCCGTTACTC
**c131181_g1**	SS/SA/AA	CGTGAGGAGTGCGTGTTAC	GATGGCTTCTTCGCTGGTC
**c158820_g1**	SS/SA/AA	TTATCCATATCTTCATCAGCAGTTG	ATACACCATCTCGTAGTCTTAGC
**c142690_g1**	SS/SA/AA	GAGACAGAGACGGAGAACAC	AGGATTGGAAGGTATGGGTAAG
**c136944_g1**	SS/SA/AA	TACCCGTTCCTTATCACCAG	TTGCCATATTCATCAGTCCTTG
**c129994_g1**	SS/SA/AA	CCGCCTCAGACACTTGC	CCTCCTTCCACTATGCTTACC
**c155944_g1**	SS/SA/AA	GTGCCGCTGAAGGAAGAG	ATGACAGATGAATTTGGAAGACC
**c147532_g1**	SS/SA/AA	GATTCCTGTGGTAATAGATACTTCC	GCATCGCTCCGTGTGAC
**c154060_g1**	SS/SA/AA	ATCCGCATTGTTCCTGAGAG	ACCTTGACCTGTTACCACTG
**c143182_g1**	SS/SA/AA	AAGGACAGATGAGTATGGAAGG	CATAACCGTGTGACCATTGAC
**c159684_g1**	SS/SA/AA	GCATCACCTCCACCACAG	CAACAGTCACCAACGCTAAC
**c153486_g1**	SS/SA/AA	CTGGCAGTGGTAGCGAATC	TGACGGTGGACGGAGAC
**c140794_g2**	SS/SA/AA	CACCTCCACCAATCTCCAC	GCCTGACGCTATACCACTG
**c141615_g1**	SS/SA/AA	GTGTGCTGATGCCATTGAC	TCCTCCAGTGCCAGTTCC
**c155531_g1**	SS/SA/AA	TTCTCCTTGGTTCCTCTTGG	ACACTTCTTGCTCCTCTGC
**c156064_g1**	SS/SA/AA	CAGCGTGACAGTGCCTTC	TCTGCCGATGACAATCTTATGG
**c157952_g1**	SS/SA/AA	TTAGAGGTCGGAATGTGATAGTC	GCAATGGATGGCGGAGAAC
**c142932_g1**	SS/SA/AA	CACATCACGCCTATGGTTATC	ACGGTATGAATTATCAGAGCAC
**c149680_g2**	SS/SA/AA	GCCGATTTGAAAGTTGGTATC	TGGTTAGGTCTGTAATGATTGC
**c159910_g1**	SS/SA/AA	TGTCTCCTCTGGGCTTGG	GAACTTGTCTGTCGCATAATCC
**c143869_g1**	SS/SA/AA	GCTCTATAATGACATCGCACTG	GACCATCAAACCTCCTTTAACG
**c138488_g1**	SS/SA/AA	TGGAGGAGACGAAGAACAAG	GTAAATGAAGCGATGAGTAAATGC
**c159244_g8**	SS/SA/AA	TCTCCAGAGTGTGACATTGC	GCTCCCTGCTGTATTATTTCC
**c130832_g3**	SS/SA/AA	GCTTGAGGTTTGTGCTTGATG	TGAATGCTACAGTTCCCAGTG
**c130187_g2**	SS/SA/AA	ATCCATTGAAATTCCCTGTAAGTG	TTCCAAGACCGTGTAAGTATCC
**c132634_g2**	SS/SA/AA	GACCTCTTACAAGTATCAACGC	GAGCCGACATCAATAAATCTGG
**c158607_g1**	SS/SA/AA	AATGGGATGTGGATGGATGTC	TTGCTGAGTGGATGCTAACC
**c136339_g1**	SS/SA/AA	ATTGTCGCATTGTTGTTTAGTTG	GGTCCATAGTTCGTTATTCCATC
**c139716_g5**	SS/SA/AA	GCAACAACGATAACAAGAACTG	GGGAAGACAAACACACTACAAC
**c134472_g1**	SS/SA/AA	CTAGAAAGGATGACAACGGAAG	AACAACGCAAGAGACAAGC
**c124714_g1**	SS/SA/AA	TCCAGCACAAGGCAGAAAC	AATGTCGGATGAAAGAAGAAAGG
**c92260_g1**	SS/SA/AA	CCCACAGATGTAAATCCCAATG	ATCCAGCAAGTTCAAATAAGACC
**c141330_g1**	SS/SA/AA	GTTTCTGGATGAAGTTCGTGAC	ACTACAACATAAACAACAAGAGAGG
**c140110_g1**	SS/SA/AA	ATGCTGGGAGTGTATAATTTAGTG	TGATCCTTATTGTCCTGTAATGAAC
**c151842_g1**	SS/SA/AA	CCAGCCACAACCACCAAG	GGACCAGCGACCCTACC
**c151092_g2**	SS/SA/AA	ACCAATGTAACTACGCTTATACTG	CTTGTTGCTAAATGACTTGAATCG
**c156875_g1**	SS/SA/AA	GAAGGAGGTAAGACGACGATAG	ATAGTGTGGCGAGACAATGG
**c134894_g1**	SS/SA/AA	TGCTTTCTGTGGTGATTCTTATG	GCCTCTGCCTTACTGTGC
**c136856_g1**	SS/SA/AA	CAGTGCCCATCCAATTCATC	CAAGGTGTCTCGTTCATTTAGG
**c149680_g1**	SS/SA/AA	ATTATGTGTAGGTGAGAGGTAGAC	AATGAGGTGTATTGTGCTTGC
**c135268_g1**	SA	ATGCGGGTCTTGTTGTAGC	GTGAAGTCTGTTGGAAGTTGTC
**c145530_g1**	SA	ATCCACTCAGCAGCAAAGG	GGCAGCAAAGCAGAATAAGC
**c145530_g1**	AA	ACATCCACTCAGCAGCAAAG	GGCAGCAAAGCAGAATAAGC
**c133300_g1**	SA	AAGAAGAATCGGTCCACACTC	TCACTGCCCTTATATTACTATTTCG
**c147741_g4**	SA	CCCATTTAGCGAGCGTTTG	TTCAGCACCGTCATTGTTTAC
**c140648_g3**	SA	TGTAACTATTGGCATTCCTCTTTG	TCATCTGTTGTCCGTCATCC
**c160088_g1**	SA/AA	AAGGCTACCACGATGATTAGAC	GATAGAACAGAACTACAACCAACC
**c144842_g1**	AA	GGCATACCAACAACTTTATCTTTAC	CGATGACGATATGAACACGAG
**c136028_g1**	SS/AA	CCATCTCATCCTCATCTCCATTC	ACAGCCCTTCCTCATTCAAAC
**c146400_g2**	SS/SA	TTGTGTGCTGCTTTGATTTCC	CACCTCAGGCTGTTGTTGG
**c131431_g1**	SS/SA	AATACGCCAATTTACAATAAACAGC	ACAATGAGGACAAACTACAGAATAC
**c146618_g2**	SS	TCAGACTACATCCAGCCAAATC	GCCACGAACGCAGAAGAG
**c146618_g2**	SA	GCCTGTGTACTATAATAGCCATTTG	GCCACGAACGCAGAAGAG
**RPS5**	SS	CCTTGATTGCTGCTACCTCTG	GCTGTTGGGAATGGAAATGG
**EF1α**	SA	ATGCACCAAGGCTGCACAGAAAG	TCCGACGTATTTCTTTGCGATGT
**EF1β**	AA	CCCAGGCAGATGCTGTTGT	GATGAGGGCGGGTTTCTT

## Results

### Sequencing and *de novo* assembly

**Fig 1:**
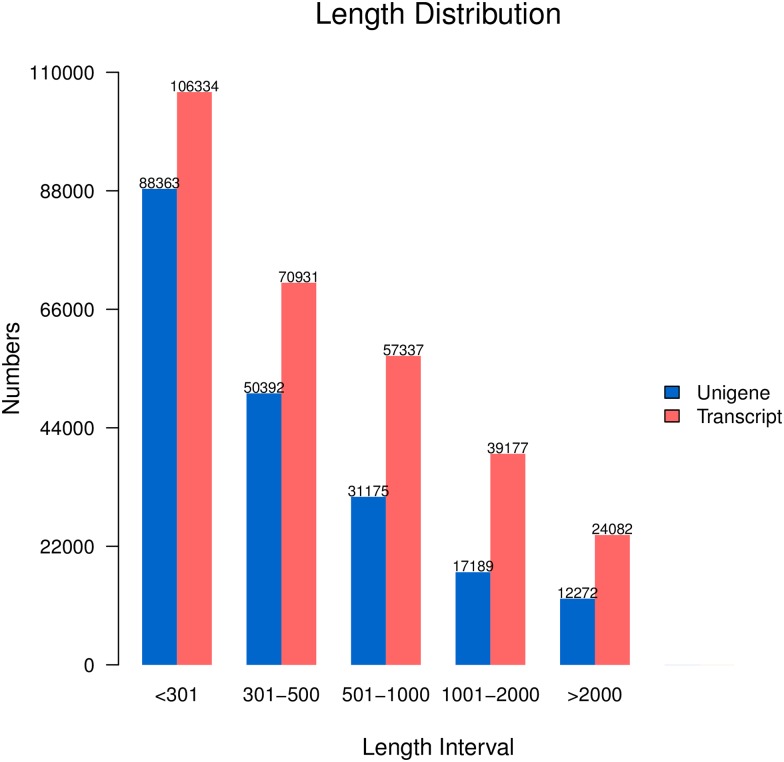
The length distribution of unigenes and transcripts.

A total of 285.62 million raw reads were obtained from the gills of SA (control + treatment) with 150 bp paired-end Illumina sequencing (S1 Table). After quality trimming and adapter clipping, a total of 280.38 million high quality reads were retained. Following removal of redundant sequences 199,391 unigenes were obtained by *de novo* assembly using the Trinity software (Table 2). Taking into account gene-splicing, 297,861 transcripts with an average length of 644 bp were described (Table 2). The length distribution of unigenes and transcripts are shown in Fig 1. The assembled transcripts had coverage of 69% of the total genome, indicating credibility of the assembly. All the sequences with the raw reads were deposited in the NCBI Sequence Read Archive (SRA) under the Accession number SRP070594.

**Table 2:** Length distribution of unigenes and transcripts.

	Min Length	Mean Length	Median Length	Max Length	N50	N90	Total Nucleotides
**Transcripts**	201	762	394	48230	1324	284	227018533
**Unigenes**	201	644	328	48230	1070	253	128406927

### Gene annotation and KOG assignment

The 199,391 Unigenes were annotated using 7 different databases by employing E-value cut-offs as the criterion for significant hit. Of these unigenes, 57,304 (28.73%) showed significant BLASTx matches in the nr database and 58,967 (29.57%) showed significant matches in the nt database (Table 3). The detailed number and percentage of annotated unigenes using various databases are provided in Table 3. Overall, nearly 40% of the unigenes could be successfully annotated by at least one of the 7 databases utilized. The top five species showing similarity with the assembled hybrid genome were *C*. *gigas* (50,037 unigenes; 87.3%), *Vitrella brassicaformis* (568 unigenes; 1.0%), *Lottia gigantea* (386 unigenes; 0.7%), *Acanthamoeba castellanii* (300 unigenes; 0.5%) and *Strongylocentrotus purpuratus* (291 unigenes; 0.5%) (S1 Fig). The high similarity of the hybrid genome with that of *C*. *gigas* can be attributed to the availability of a complete high quality genome for this species and the fact that it is one the congeneric parents of the hybrid [37].

**Table 3:** The number and percentage of annotated unigenes using 8 databases.

Database	Number of Unigenes	Percentage (%)	E-value<
**Nr (NCBI non-redundant protein sequences)**	57304	28.73	1e-5
**Nt (NCBI non-redundant nucleotide sequences)**	58967	29.57	1e-5
**KO (KEGG Ortholog database)**	13207	6.62	1e-3
**Swiss-Prot (A manually annotated and reviewed protein sequence database)**	26579	13.33	1e-5
**Pfam (Protein family)**	39069	19.59	0.01
**GO (Gene Ontology)**	39145	19.63	1e-6
**KOG (Clusters of Orthologous Groups of proteins)**	18122	9.08	1e-3
**Annotated in at least one Database**	82968	41.61	

KOGs is a tool to identify orthologous and paralogous proteins in eukaryotes and annotate the proteins according to their functional categories [38]. Of the 199,391 defined unigenes, 18,122 were successfully annotated (Table 3) and classified into 26 KOG categories. Amongst the various KOG categories, the signal transduction mechanisms (T, 18.8%) was most highly represented, followed by Post translational modification, protein turnover and chaperones (O, 10.9%). The statistical analyses of the enriched KOG functions allowed us to tease apart specific functions that were affected in each salinity model.

### Analysis of differentially expressed genes

66 differentially expressed genes were identified with the filtering criterion of log_2_ fold change (low salinity group/control group) ≥ ±1 and adjusted *p*-value < 0.05. Of these, 35 genes were up-regulated and 31 genes were down-regulated in oysters maintained in low salinity conditions in comparison to controls (S2 Table). Functionally, these DEGs were found to be involved in various physiological processes including immune response mechanisms, apoptosis pathways, energy metabolism, osmoregulation and other cellular processes (S3 Table).

### Gene Ontology annotations and pathway analysis

Gene Ontology (GO) annotations were performed to assign the DEGs to the categories of biological process, molecular function and cellular component [39, 40]. Under the category of molecular function, transcripts for N-glycosyl hydrolases (GO: 0016799) were highly represented in both up- and down regulated genes (Table 4). Similarly, components of respiratory chain complex IV (GO: 0045277) were observed under cellular component category in both up- and down-regulated genes sets (Table 4). For biological process, the terms nucleoside monophosphate catabolic process (GO: 0009125); deoxyribonucleoside monophosphate catabolic process (GO: 0009159), mitochondrial electron transport, cytochrome c to oxygen (GO: 0006123) and visual perception (GO: 0007601) were highly represented in both up- and down regulated genes. (Table 4)

**Table 4:** The first 20 GO enrichment terms.

GO accession	Term description	Term type	*P*-value
**GO:0016799**	hydrolase activity, hydrolyzing N-glycosyl compounds	MF	0.000513
**GO:0009125**	nucleoside monophosphate catabolic process	BP	0.004394
**GO:0009159**	deoxyribonucleoside monophosphate catabolic process	BP	0.004394
**GO:0050144**	nucleoside deoxyribosyltransferase activity	MF	0.004394
**GO:0070694**	deoxyribonucleoside 5'-monophosphate N-glycosidase activity	MF	0.004394
**GO:0004129**	cytochrome-c oxidase activity	MF	0.007347
**GO:0006123**	mitochondrial electron transport, cytochrome c to oxygen	BP	0.007347
**GO:0016675**	oxidoreductase activity, acting on a heme group of donors	MF	0.007347
**GO:0016676**	oxidoreductase activity, acting on a heme group of donors, oxygen as acceptor	MF	0.007347
**GO:0045277**	respiratory chain complex IV	CC	0.007347
**GO:0015002**	heme-copper terminal oxidase activity	MF	0.008339
**GO:0007601**	visual perception	BP	0.008446
**GO:0030551**	cyclic nucleotide binding	MF	0.008446
**GO:0030553**	cGMP binding	MF	0.008446
**GO:0050953**	sensory perception of light stimulus	BP	0.008446
**GO:0098803**	respiratory chain complex	BP	0.011175
**GO:0000702**	oxidized base lesion DNA N-glycosylase activity	MF	0.011714
**GO:0006285**	base-excision repair, AP site formation	BP	0.011714
**GO:0008534**	oxidized purine nucleobase lesion DNA N-glycosylase activity	MF	0.011714

MF, means molecular function; BP, means biological process; CC, means cellular component.

In addition to GO analysis, the DEGs were mapped to various pathways based on KEGG analysis. In total, the DEGs were mapped to 25 pathways as described in KEGG. These pathways include those related to: immune mechanism- NOD-like receptor signaling pathway (ko04621), MAPK signaling pathway (ko04010), Endocytosis (ko04144), phagosome formation (ko04145) and so on; cellular proliferation- TNF signaling pathway (ko04668), apoptosis (ko04210); protein synthesis- protein processing in endoplasmic reticulum (ko04141); energy metabolism- glycan degradation (ko00511) (Table 5).

**Table 5:** All enrichment pathways.

ID	Pathways	Input number	Background number
**ko04621**	NOD-like receptor signaling pathway	2	73
**ko04064**	NF-kappa B signaling pathway	2	82
**ko04668**	TNF signaling pathway	2	91
**ko04210**	Apoptosis	2	99
**ko05222**	Small cell lung cancer	2	107
**ko04120**	Ubiquitin mediated proteolysis	2	232
**ko04910**	Insulin signaling pathway	2	243
**ko04510**	Focal adhesion	2	296
**ko00511**	Other glycan degradation	1	46
**ko05200**	Pathways in cancer	2	309
**ko04514**	Cell adhesion molecules (CAMs)	1	53
**ko04141**	Protein processing in endoplasmic reticulum	2	335
**ko03060**	Protein export	1	73
**ko04612**	Antigen processing and presentation	1	80
**ko05162**	Measles	1	122
**ko04330**	Notch signaling pathway	1	123
**ko05134**	Legionellosis	1	138
**ko05110**	Vibrio cholerae infection	1	155
**ko04915**	Estrogen signaling pathway	1	159
**ko05164**	Influenza A	1	190
**ko04010**	MAPK signaling pathway	1	191
**ko03040**	Spliceosome	1	250
**ko05169**	Epstein-Barr virus infection	1	261
**ko04144**	Endocytosis	1	264
**ko04145**	Phagosome	1	280

### Validation and analysis of DEGs in SS, SA and AA by qRT-PCR

**Fig 2:**
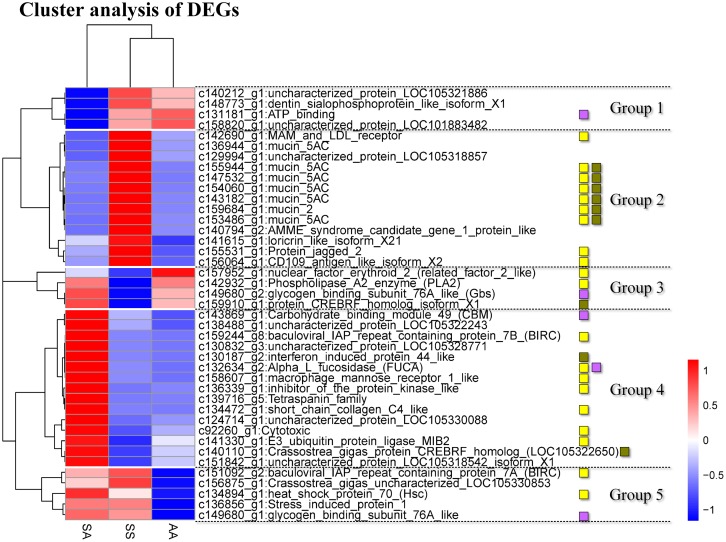
Cluster analysis of DEGs in SS, SA and AA oysters based on their relative expression level as determined by qRT-PCR. Blue represents lower expression, and red represents higher expression. Each column represents a comparison between low salinity samples and control samples for each species. Each row represents a gene. The yellow square represents immune- or apoptosis-related gene, the green square represents genes involved in osmotic regulation, and the purple square represents genes involved in energy regulation. The 41genes were classified into five groups.

Among the 66 DEGs, 59 genes were annotated by data base. For each of these 59 genes, appropriate qRT-PCR primer pares were designed for the SS, SA and AA individuals. Using the cDNA extracted from the gills of SS, SA, and AA oysters, the transcript level of 45 genes in SS, 50 genes in SA, and 45 genes in AA were successfully analyzed by qRT-PCR. Of these genes assayed, 41 genes were shared among all the three groups (Fig 2, S4 Table). Gene expressions in the hybrids SA under low salinity stress were analyzed and compared with the RNAseq data. 92% of the genes showed high correlation with RNA-seq in SA oyster gills (R = 0.928, P = 1.76E-20) (S4 Table). Thus the qRT-PCR analysis confirmed our RNAseq results indicating the reliability and accuracy of our high-throughput analysis. Finally, some of the 41 genes in the SA hybrids showed differential expression with respect to the parental oysters. These results build confidence in our Trinity based transcriptome assembly as well.

## Discussion

Recently, high-throughput sequencing has been increasingly applied to study a wide spectrum of model and non-model animals. The transcriptomes of several oyster species such as pacific oyster (*Crassostrea gigas*) [41], eastern oyster (*Crassostrea virginica*) [42], Fujian Oyster (*Crassostrea angulata*) [43] and HongKong oyster (*Crassostrea hongkongensis*) [44] have been characterized by high-throughput sequencing. In this study, we have compared transcriptomes of *C*. *sikamea* (SS) ♀ × *C*. *angulata* (AA) ♂ hybrids (SA) maintained in low salinity stress (10 ppt) and natural seawater with optimal salinity as controls (33.5 ± 0.3 ppt). Osmoregulation is a complex physiological process that allows oysters to exist at varying salinities in their natural habitat. RNA-Seq technology is an important tool which can reveal the mechanism of osmoregulation by identifying the genes involved in the process. It is worth mentioning that mantle, rather than shell, is the key tissue of marine shelled mollusk that helps the organism to cope with the osmotic disbalance in the surrounding environment by tightly sealing the mantle cavity and temporarily inhibiting water-salt exchange [45]. Deliberate opening of the shell by force (chipping away a part of shell edge of oyster [29] or inserting corks between the shells [46]) under low salinity conditions exerts a sudden acute osmotic shock not encountered by the organism in natural conditions. To circumvent this problem, we used a method of lowering the salinity of the seawater gradually by adding freshwater to the culture tank. This helped us in preventing osmotic shock to the oysters while subjecting them to osmotic stress. Such gradual decline in salinity is also observed in estuaries as a result of heavy rainfall or mixing of seawater of different salinities. Additionally, in contrast to the osmotic stress studies involving fish, shrimp, and deliberately opened shellfish, the intact shells in our study protected the oysters from sudden osmotic stress. Thus, unlike previous studies where low salinity stress was induced in various bivalves by human intervention, our experimental design reflects natural estuarine saline stress conditions. We believe that the 66 DEGs identified as low salinity regulated genes are more likely to show change under natural conditions of salinity stress. We used RNAseq to elucidate the expression profile of the genes and pathways related to immune mechanism, apoptosis, energy metabolism and osmotic regulation in oyster hybrids following gradual changes in ambient salinity.

### Immune mechanism

Oysters lack adaptive immune system and rely solely on various innate immune response mechanisms [47]. Of the various organs, gills play a key role in the innate immunity of oyster [48–52]. We found that nearly 40 percent of the 59 annotated DEGs were immuno-regulatory genes. The induction of immune genes under low salinity stress has been reported in other oysters thus supporting our observation [28, 53]. These findings also indicate that hypotonic environment can induce an immune response in bivalves.

The mitogen-activated protein kinases (MAPKs) play important roles in response to anoxia, freezing and osmoregulation [54]. The stress-activated MAPKs, comprising of JNK and p38, regulates key cellular event, such as cell migration and phagocytosis, and contribute to the innate immune system of an organism [55]. In this study, the MAPK signaling pathway (ko04010) was found to be activated in hybrid oysters maintained under low salinity stress, indicating induction of the innate immune response. Similar results have been reported in the hemocyte of the white shrimp (*Litopenaeus vannamei*) under salinity stress [16], further supporting our observation.

NLRs (NOD-like receptors) are a family of proteins which can recognize intracellular ligands and play a crucial role in innate immunity [56, 57]. There are 22 NLRs in humans, while mice has at least 33 [58]. The earliest identified and best characterized NLRs are NOD1 and NOD2, which has the ability to sense cytosolic bacterial peptidoglycan fragments evading detection by phagosomes. This interaction activates NF-κβ and MAPK pathways, thereby regulating cytokine production and apoptosis [58, 59]. Previous study have found that the expression of three essential components (apoptosis-associated speck-like protein, Nod-like receptor protein 1, caspase 1) of NOD-like receptor protein are altered under high osmotic stress [60]. Additional evidence suggests that osmotic stress activates TAK1 gene. The kinase of TAK1 (TAK1 kinase) is an indispensable intermediate of NOD-like receptor signaling pathway [61]. In this study, the NOD-like receptor signaling pathway (ko04621) was found to be enriched in marine bivalves under low salinity stress indicating the induction of this pathway under hypotonic conditions.

E3 ubiquitin-protein ligase is an essential part of the protein degradation machinery [62, 63]. Recently, it has been implicated to play a role in osmoregulation and innate immunity in shrimp (*Penaeus monodon*), where the enzyme has been found to be up-regulated under low salinity stress [64]. In our study, a clear induction of the E3 ubiquitin-protein ligase was noted under low salinity stress, further supporting the enzyme’s role in innate immunity. Thus overall, we see an induction of MAPK activity, NOD-like receptor signaling pathway and ubiquitin conjugating enzyme under salinity stress. Although NOD-like receptor signaling pathway and MAPK signaling pathway play a significant role in osmotic stress management, the involvement of ubiquitin conjugating enzymes in salinity stress needs to be investigated further. A similar simultaneous induction of these three pathways have been observed in Tilapia (*Oreochromis mossambicus*) under salinity stress [65].

The cellular immunity, which includes nodulation, encapsulation, and phagocytosis, are an important part of the innate immune system of the invertebrates [66]. Phagocytosis is a fundamental cellular process that serves multiple functions in host defense [67]. The phagocytic process begins with pathogen recognition followed by the binding of the pathogen derived ligand to cell surface receptors [68]. The pathogen is then engulfed and internalized by phagosomes, which forms a part of the endocytotic machinery called phago-lysosomal pathway [16, 68]. This phenomenon is associated with production of reactive oxygen species (ROS). Also known as ‘respiratory burst’, cells display strong anti-microbial activity during this phase [69]. In addition, phagocytes supplement its immune responses by releasing lysozyme and elastase resulting in the elimination of pathogenic invaders [16, 70].

We found that the gene Sec 61 Alpha-1 (SEC61), usually associated with phagosome formation, was significantly down-regulated in oyster hybrids under low salt stress. SEC61 is known to participate in translocation events from the phagosome lumen to the cytoplasm [71]. Thus, our result suggests that low salinity stress would affect cellular immunity via the translocation events. On the other hand, Hsc70, a gene involved in endocytosis (K03283), showed significant up-regulation under low salinity stress. Previous studies have shown that in Hsc70 mutants, the uncoating of vesicles was delayed thus prolonging the life-time of endocytic vesicles in the cell [72, 73]. Our results suggested a reduction in endocytosis in the hybrid oysters under low salinity stress. Additionally, mucin-5AC (MUC5AC) and jagged-2 (JAG2) genes, involved in mitochondrial electron transport (GO: 0006123), showed significant down-regulation under low salinity stress suggesting a reduction of oxygen-independent reactivity.

Humoral defense includes production of antimicrobial peptides, induction of lectin synthesis, and activation of the prophenol-oxidase (proPO) system [74]. Phospholipase A2 enzyme (PLA2) has a crucial role in liberating free fatty acids and lysophospholipids from membrane phospholipids, thereby initiating the production of biologically active lipids, which mediate inflammatory reactions in mammals [75]. *PLA2s* have been reported to promote bacterial killing by degrading membrane phospholipids [76, 77]. In the current study, a phospholipase with significant homology to *C*. *gigas PLA2* was expressed at a higher level in the gills from low salinity stressed hybrid oysters, compared to the control group. Presumably, the paralogs of *PLA2* are involved in humoral immune reactions during low salinity stress condition in oysters. In *L*. *vannamei* the prophenoloxidase-activating enzyme 1a gene was reportedly up-regulated in low salinity stress, providing evidence that low salinity environments induce humoral immune responses in crustaceans. [16]

### Apoptosis

Apoptosis is essential for the development and maintenance of cellular homeostasis of the immune system [29, 78]. The baculoviral IAP repeat-containing proteins (BIRCs), are inhibitors of cell death which act by binding to active caspases [79]. Earlier studies suggest that in addition to inhibiting apoptosis, IAPs are also involved in signal transduction and cell cycle regulation [80]. This gene has been shown to be a part of the broader network of NF-κβ signaling pathway (ko04064), TNF signaling pathway (ko04668), Apoptosis (ko04210) and NOD-like receptor signaling pathway (ko04621). Our data shows that the genes encoding BIRC are up-regulated in oyster hybrids under low salt conditions, in addition to a noted down-regulation of all these allied pathways. It is interesting to note that we have found induction of genes that inhibit apoptosis and a general repression of apoptosis in pathway analysis. We think the suppression of apoptosis leads to better cell survival in low salt conditions and might be one of the key reasons for the better adaptability of SA hybrids under osmotic stress making them an euryhaline species [20]. Our findings are in accordance with previous studies in *L*. *vannamei* and *Crassostrea gigas* [16, 29].

### Energy metabolism related genes

Energy in the form of ATP is required for both osmoregulation and cellular homeostasis [81, 82]. Under low salinity stress, proteins and free amino acids from the body tissue or ingested food are usually catabolized providing energy and the ions required to maintain the osmotic pressure [83].

Carbohydrate-binding module (CBM) is a protein domain which shows carbohydrate-binding activity and is found in carbohydrate-active enzymes (for example glycoside hydrolases). [84]. Alpha-L-fucosidase (FUCA) is a hydrolase enzyme [85] which has been recently reported to have transglycosylation properties in both invertebrates and vertebrates [86]. The phosphorylated glycogen-binding subunit (Gbs) of the protein phosphatase 1 remains largely bound to glycogen forming the core of glycogen granule. Protein phosphatase 1 plays a pivotal role in glucogen metabolism and aids the conversion of glucose to glycogen [87, 88]. Normally these three genes require ATP for their function and might involve the ATP binding protein. In our study, the genes encoding CBM49, FUCA, Gbs-76A and ATP binding protein were found to be induced in hybrids maintained under low salt conditions. Thus, low salinity can also affect the functionalities of enzymes involved in energy metabolism. A similar finding was reported in a case study of *L*.*vannamei* exposed to different stress levels of salinity [40].

### Osmoregulation-related genes

Oysters are osmoconformers having the capacity to regulate cell volume over a wide range of external osmotic concentrations [89, 90]. However, they lack the ability to osmoregulate their extracellular fluid [89, 90]. Salinity fluctuations thus result in the release of osmotically active solutes (osmolytes) in order to maintain osmostic balance [90]. The osmolytes include inorganic ions and free amino acids (FAA) [89]. Our study indicates the involvement of protein coding genes having various functions in osmoregulation. A membrane transport protein (or simply transporter) is involved in ion transport across cell membranes. We found significant upregulation of a transporter gene, encoding transient receptor potential cation channel protein, under low salinity stress in the gills of the SA oysters. Proteolysis catalyze the hydrolysis of proteins into smaller polypeptides or amino acids. In this study, a disintegrin and metalloproteinase (ADAM) gene was found upregulated (535-fold) under low salinity stress in the gills of SA. This indirectly proves that inorganic ions and FAAs may both participate in osmotic regulation in SA under low salinity stress. Our results were similar to the proteomic analysis data obtained by Meng et al. under low salinity stress [91]. Mucin genes encode mucin monomers which are synthesized as rod-shaped apomucin cores that undergo post-translational modification by abundant glycosylation [92]. As a result of dense ‘sugar coating’, mucins show high water-holding capacity and resistance to proteolytic cleavage [93]. Our study revealed the downregulation of seven mucin genes under low salinity stress suggesting that low salinity stress may promote proteolysis. This finding is consistent with the upregulation of the metalloproteinase (*ADAM*) gene observed in the present study.

### Differential expression pattern in SS, SA and AA

Expression patterns of the 41 genes in the gills of SS, SA and AA were classified into 5 groups (Fig 2). Notably, 7 mucin genes, which were placed in group 2 were upregulated in SS under low salinity stress but downregulated in the AA and SA oysters (Fig 2, S4 Table). As already mentioned, mucins show high water-holding capacity and resistance to proteolytic cleavage [93] and downregulation in SA and AA oysters. Suprisingly, an opposite trend was observed in mucin gene expression pattern in SS oysters. This is an interesting finding which shows that the hybrid inherits the traits from one particular parent (mucine gene expression patter of AA oyster) that are involved in osmoregulation. Our observation that different species exhibit different types of osmoregulation in response to low salinity stress is consistent with previous experiments, wherein rainbow trout and fathead minnow, exhibited different sensitivities to osmoregulation toxicants [94].

Furthermore, all 15 genes from group 4 showed a higher expression in the SA hybrids in comparison to the parental SS and AA oysters, when maintained in a hypotonic condition (Fig 2, S4 Table). Among the 15 genes, 7 immune- or apoptosis-related genes were identified. Similarly, in group 3, 2 genes were activated in SA hybrids in comparison to SS oysters under low salinity stress (Fig 2) while in group 5, 2 genes were activated in SA hybrids in comparison to AA oysters. These results are consistent with previous observations that fluctuations in salinity often compromises the immune system in mollusks and reduces immune defenses of the stressed clams [30, 95]. Studies have shown significant changes in ROS production and antioxidant enzyme expression or activities in marine organisms upon exposure to low salinity [96]. A total of 11 immuno-regulatory genes were present in the groups 3, 4 and 5. These genes in the gills of SA showed much higher expression than SS or AA, suggesting that the immune-regulatory genes in SA show higher response under low salinity as compared to AA and SS.

As mentioned earlier, CBM49, FUCA and Gbs76A require ATP for their function and might involve ATP binding protein (please see ‘Energy metabolism related genes’). The increase in expression level of these three genes in the gills of SA were higher than either SS or AA or both, under low salinity stress. It is likely that these proteins involved in energy metabolism respond to low salinity more strongly in SA than in SS and AA. Thus it can be inferred that genes relative to immune mechanism, apoptosis, osmoregulation and energy metabolism are differentially expressed in SS, AA and SA oysters when subjected to osmotic stress. However, further studies are necessary to elucidate the immune response mechanisms in oysters under low salinity stress conditions.

## Conclusion

In general, our work represents the first report of immune response in *C*. *sikamea* (SS) ♀ × *C*. *angulata* (AA) ♂ hybrids (SA) upon exposure to low salinity stress utilizing the next generation sequencing technology. A total of 66 unigenes were significantly altered after 16 h of continuous low salinity stress. In our study, we identified a number of up and downregulated genes involved in diverse pathways including immune mechanism, apoptosis, osmoregulation and energy metabolism. These results highlight a complex network of immunological and metabolic pathways in the gills of oyster hybrids. Our findings would facilitate future research on immune response mechanisms, apoptosis, osmoregulation, and energy metabolism in oyster hybrids under low salinity stress to a considerable extent.

## Supporting information

S1 Fig The top five species showing similarity with the assembled hybrid genome. (DOCX)

S1 Table Summary of Illumina expressed short reads production and filtering. List providing information on reads number.
(XLSX)

S2 Table Differentially expressed unigenes in low salinity group. List of read counts and Log_2_ Fold Change of all DEGs from hybrid oysters in response to low salinity stress in comparison to control condition.
(XLSX)

S3 Table Gene function. Lis of gene function of all the DEGs from hybrid oysters in response to low salinity stress in comparison to control condition.
(XLSX)

S4 Table Relative gene expression by qRT-PCR. List of relative gene expression from hybrid and parental oyster species in response to low salinity stress in comparison to control condition.
(XLSX)
