# Supplementary figures and images for: Transcriptomic analysis of Crassostrea sikamea × Crassostrea angulata hybrids in response to low salinity stress
Source: PLoS One. 2017 Feb 9;12(2):e0171483. doi: 10.1371/journal.pone.0171483 (PMC5300195; doi:10.1371/journal.pone.0171483)

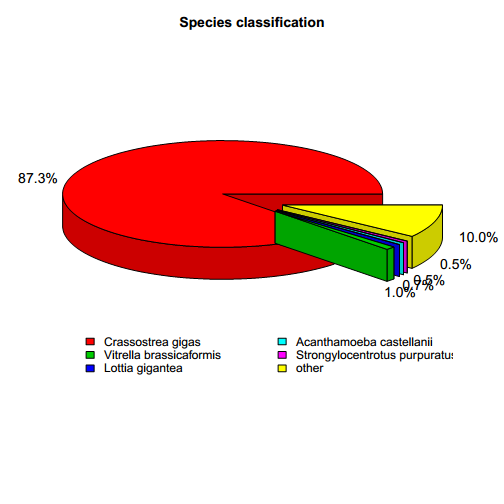


S1 Fig. The top five species showing similarity with the assembled hybrid genome.

Supplement: S1 Fig — (DOCX) [file pone.0171483.s001.docx]
